# Fifty Years in the Laboratory; or the Evolution of the Dental Laboratory as I Have Seen It

**Published:** 1895-08

**Authors:** L. P. Haskell

**Affiliations:** Chicago, Ill.


AMERICAN JOURNAL

—OF—

DENTAL SCIENCE.

Vol. xxix. Baltimore, August, 1895. No. 4.

ARTICLE I.

### FIFTY YEARS IN THE LABORATORY; OR THE EVOLUTION OF THE DENTAL LABORATORY AS I HAVE SEEN IT.

L. P. HASKELL, CHICAGO, ILL.

What I shall have to say to-day will be largely personal history, as it will relate to my personal experiences of half a century.

Were the graduate of the dental college of to-day to be placed in the dental laboratory of fifty years ago, and attempt to construct a denture as then made, he would throw up his job, for such is the great advance made in tools and appliances, materials and methods; he would consider it almost impossible to construct a denture by the old methods.

In January, 1845, I entered on my duties in a dental office in Boston, or rather its suburb, Chelsea. At that time no one considered it necessary to obtain his dental education in a college; in fact, the only one in existence was the Baltimore, then in its infancy, and which remained solitary and alone for many years. A large proportion of dentists picked up their dental knowledge here and there, with the doors of the operating rooms and laboratories largely closed against them; no dental journals; no text-books crude; no dental societies, and very little intercourse between the members of the profession.

There was, however, an absolute necessity for a knowledge of working metals, as there was no dental depots to resort to for supplies of gold and silver plate and solder, so the dentist must learn to melt gold, refine his old plates and filings, roll his plate, and make solders.

As the average dentist was not successful in saving teeth, especially if badly decayed or abscessed, the necessity arose for cultivating skill in the construction of dentures, and as they were all of metal, it was not every one who made a success of it.

In taking impressions common beeswax was the only material used. The impression-cups were made by the tinsmith. My preceptor was the first dentist in New England, so far as I am aware, to take an impression in plaster, but it was years before it came in general use, and the material was of an inferior quality.

My first experience in the making of dies was in the use of tin. To do this the counter, or as then termed, the female die, was made first, by plunging the model, perfectly dry, in the lead. The removal of the model necessitated its destruction. A rim of several thicknesses of paper was placed around it and the tin poured in it. After a time, type metal, zinc, and brass were used. After I had been in practice for myself two years, I was led to try what was then known as "Babbitt's anti-friction metal," then just introduced into this country for machinery bearings. This, with some slight modifications I have used ever since, finding it the only alloy that has all of the requisite qualities for a dental die, and has greatly simplified the fitting of plates.

At this time, or at my entrance on the stage, spiral springs were an absolute necessity for retaining an upper plate in place, and so continued for many years afterward in the practice of many dentists, and I am informed are still considered a necessity in England. My preceptor was the first dentist in New England who first constructed a suction-plate, in the year 1844. If any one knows of any attempts prior to this I would be pleased to be informed.

The impression was taken in wax, the die made of tin, and the result very gratifying. To test the suction, he soldered a hook to the centre of the plate, placed it in the mouth, told the patient to suck it up, attached a wire to it, and suspended thereto an ordinary water-pail filled with water, and placed other weights on that. These plates were fitted to the palate, the "air-chamber" known as the Gilbert, being introduced later, came in general use; and still later what was known as the Cleveland or soldered chamber. I have, however, dispensed with all forms of air-chambers for twenty-five years, deeming them superfluous, in fact, in many cases highly detrimental.

The carved bone and ivory teeth had been displaced by porcelain teeth. The first I used of these were Alcock's, made in Philadelphia. These were followed by Stockton's, which were an improvement on the former. A few years later his nephew. S. S. White, who obtained his knowledge of the business from his uncle, established a factory under the firm name of Jones, White & McCurdy, and soon produced an article far superior to any thing previously made. In fact, as regards translucency and delicacy of coloring superior to any thing now made. But afterward, to increase the strength, these features were in a measure lost, as the increase of silex and clay made them more opaque.

Up to this time all dental goods were sold by druggists. This firm entered in the manufacture of other dental goods, and established dental depots in Philadelphia, New

York and Boston. They kept no supply of plate and solder, and the dentist, if he did not make it himself, resorted to the manufacturing jeweler for his supply.

Every tooth was soldered to the plate, and skill was required in selecting, grinding to the plate, backing and soldering full sets of gum teeth, an experience which few practitioners of to-day have had.

The use of gas for soldering was not known till a later date, and the alcohol lamp and mouth blow-pipe were the appliances then used.

Lathes were of rude construction. Emery wheels, and not coarse at that, were used for grinding. What a contrast to the modern dental lathe, electric motor and carborundums. The appliances for finishing were few and rude. Think of the various appliances used on the dental engine for finishing and doing fine work.

At the time I commenced the business, carved block teeth were being introduced. These, of course, were carved for each case, and for eleven years I was engaged in carving and mounting for our own practice and for the profession in the New England States.

My first experience in the laboratory was the preparation of the material, calcining, breaking in small pieces and grinding in a quartz mortar, quartz and felspur to an impalpable powder. These, together with clay or kaolin and coloring matter of the oxids were then mixed ready for carving. Oxid of gold and tin were used to produce the flesh color of the gum enamel.

Then there was the management of the furnace, constructed of fire-brick, and soap-stone covers and stoppers, with two or more muffles. The baking was done in the latter part of the day, and running sometimes in the night quite late. The work was not examined till morning. Carving, biscuiting, trimming, putting in the platina pins, enameling and baking of two sets was considered a day's work.

A considerable number of dentists learned to do this, but with varying success, as it required skill and taste which all did not possess. I instructed the fathers of quite a number of the present generation.

In 1852 an agent of John Allen's came to Boston to sell office-rights for the use of his new patent, since known as Allen's Continuous Gum Work. We purchased an office-right for $150, and have continued the use of it to the present time. For full sets this was the greatest advance made in prosthetic dentistry, and remains to-day the most perfect denture ever devised, for strength, durability, naturalness of appearance, healthfulness to the membrane and cleanliness.

Soon after the introduction of vulcanized rubber, dentists in various parts of the country made a combination of continuous gum and rubber, which had its run for several years, and was then abandoned. One dentist who made it for several years was afterward asked his opinion of it, and replied, "It was like the devil's tail painted blue, more ornamental than useful." The serious objection to it was like some other combinations, it made the repair too expensive.

Like some other "discoveries" this method has been discovered, or re-discovered, several times since, patented and presented to the profession as something original. The lesson to be learned from this is, that no denture should be put in the mouth which cannot be readily repaired at a fair expense.

I think it was in the year 1852, a dentist named Slayton came to Boston to introduce a method of inserting dentures on gutta-percha. I have in later years made use of it in what I call "emergency" work. As for instance, a physician came to my office at 5 p. m., had several teeth extracted, impression taken, a plate of gutta-percha formed on the model, teeth arranged, and fastened to a rim of the same material by heat, and the gum contoured with a hot spatula; at 7.30, two and a half hours, he was eating his dinner with his new teeth at a restaurant. Also a temporary lower plate, made in the same manner in three hours, while the patient waited, lasted one year.

Also about the year 1852, a dentist named Levett came to Boston to introduce an enamel for gold plates. I was wearing a small gold plate and had it enameled, but in a short time it cracked off. It convinced me that enamel which could be baked on gold plate was merely glass, and not fit to be worn in the mouth. This is also one of the "inventions" which has been "discovered" several times and found worthless.

About the same time, or a little earlier, Dr. Loomis, of Cambridge, Mass., patented the porcelain plate, which has been improved on to some extent, and is still used by a limited number of dentists. A first-class, artistic denture cannot be made by this process, and is difficult to repair.

I think about the year 1855, Dr. Blandy, of Baltimore, introduced a metal he called "cheoplasty," for making cast plates. It was similar to Watt's and Weston's metals composed of tin and bismuth,

In 1858, then in business in Chicago, associated with Dr. Allport, I made a visit to Boston, and calling on an old friend, Dr. J. A. Cummings, he took me aside and showed me, in a confidental way, a vulcanized rubber plate, for which he said he had applied for a patent. The outcome and history of which you are familiar, with some to your sorrow in the contest with Bacon.

The introduction of this material caused a retrograde movement in prosthetic dentistry, and though it has its merits, has been a detriment to the profession, for so simple are its methods that it has enabled a host of incompetent men to foist themselves on the community, and also led many of the better class of dentists to abandon a class of work far superior, simply because the work was easier, and could be done by mere novices in the laboratory.

Dr. Allport has well expressed the idea in an address before the Boston Academy of Dental Science :

"He who has but moderate ideas of symmetry, harmony of expression and color, is constantly pained by the lack of that artistic selection and arrangement of artificial teeth which serves to restore to the face the shape and expression left on it by the Creator, the absence of which in artificial dentures stamps him who should be an artist, an *artisan—a mere mechanic—a libeller of the soul—a deformer of the human face divine.* That mechanical dentistry should have very largely fallen in the hands of this inferior class of practitioners will hardly be wondered at by those who have watched the history of this branch of the practice. For so simple are the modes of attaining tolerable mechanical results with the methods now usually employed in this department, that a high order of appropriate talent is, at present, seldom found devoting much time to it."

The most serious objection to the use of rubber is found in the fact that the retention of undue heat causes constant change in the alveola process, the exceptions to which are very rare.

In the early 60's, Dr. McClelland, of Louisville, introduced a material for plates which he termed "Rose Pearl," a preparation of gun-cotton and camphor. This was followed by an improved article called Pyroxolene, which made a very handsome plate, but proved a failure; I made several plates of it. Next came celluloid, a decided improvement on the other, and which has continued in use. I used it instead of rubber for four years and then abandoned it.

One of the more recent inventions has been the "Electro-deposit plate," professedly gold, but only a silver plate with a very thin deposit of gold on its surface. It has very serious objections, and those who have used it, so far as I know them, have abandoned it.

Though aluminum has been used to some extent for plates for many years, it did not prove a success till recently, on account of the difficulty arising from the presence of specks of iron in the metal, which rusting produced holes in the plate. It makes a firm and more rigid plate than any other metal and is easily swaged. I find the secretions do not affect it at all. By using the loop-punch a firm adhesion of the rubber is obtained.

Ever since the introduction of rubber and celluloid there has been a set-back to prosthetic dentistry till the introduction of crown- and bridge-work, which has made it necessary for the dentist to learn the use of metals, so that to-day prosthetic dentistry occupies a higher plane than ever.

Dr. Land is entitled to credit for the introduction of the "jacket crown," etc. But far greater is Dr. Parmly Brown's method of porcelain crowns and bridges, which seem to me the *ne plus ultra* of prosthetic work, its two important features being the non-mutilation of teeth and showing no metal in the mouth.

The introduction of porcelain work to such an extent has necessitated the invention of gas furnaces, so that one improvement after another has developed some almost perfect furnaces. The Parker, an open-flame furnace, has done most excellent work, followed by the Downie with a platinum muffle which seemed almost perfection, till recently Land has brought out a furnace, the "Revelation," in which coal oil is used. In this furnace no bellows is needed, simply a good draft in a chimney. The high fusing bodies have been fused successfully. I am using it satisfactorily.

Though there has been great improvement in the manufacture of teeth there is still room for more. One serious fault is the unnecessary multiplication of molds, sometimes done to satisfy the whim of some dentist, but oftener because the manufactures fails to realize the real necessities of the case. In the S. S. White catalogue of teeth are nearly one hundred molds of upper plain bicuspid and molar rubber teeth! Many of them so near alike it is difficult to distinguish between them; others so unshapely it is a wonder any one finds a use for them; but the most serious fault is found in the fact that even in the longest of them there is little porcelain above the pins. In grinding to articulate, this small amount of porcelain is often ground away, or so nearly so that what is left is broken off very soon. Not only this, but the rubber gum comes so near the crown that it is unsightly. In nearly all of these teeth the pins can be placed in a position to give longer cusps. The company, however, have been making some new methods in accordance with these suggestions.

There are too many molds with too little masticating surface; too often the lingual cusps of the upper teeth are too long; they should be shorter than the buccal.

In plain upper rubber bicuspids and molars twelve variations of molds would be all that is necessary, and consider how this would simplify the selecting of teeth by the dentist or the depot clerk.

These same faults exist to a variable extent in all makes of teeth. I would very much like an expression of opinion on the subject by dentists.

In the teaching of prosthetic dentistry in the colleges there are serious faults, the reasons of which are threefold.

1st. Too much of the student's time is occupied in the lecture-room, in the endeavor to teach him methods which can never be comprehended till he sees them done or does them in the laboratory.

2d. In the laboratory he is taught in classes, and not enough individually, and these classes so large as to be unwieldly.

3d. The demonstrators are too often inexperienced men, graduates perhaps of a previous year. In no place in a college is wide experience more necessary than in the laboratory.—*Ohio Dental Journal.*

